# Isolated Joint Block Progression Training Improves Leaping Performance in Dancers

**DOI:** 10.3389/fspor.2021.779824

**Published:** 2021-12-14

**Authors:** Paige E. Rice, Kiisa Nishikawa, Sophia Nimphius

**Affiliations:** ^1^School of Medical and Health Sciences, Edith Cowan University, Joondalup, WA, Australia; ^2^Department of Health and Exercise Science, Wake Forest University, Winston Salem, NC, United States; ^3^Department of Biological Sciences, Northern Arizona University, Flagstaff, AZ, United States

**Keywords:** power, strength, joint kinetics, muscle, tendon, ankle

## Abstract

The purpose of this study was to investigate the effect of a 12-week ankle-specific block progression training program on *saut de chat* leaping performance [leap height, peak power (PP), joint kinetics and kinematics], maximal voluntary isometric plantar flexion (MVIP) strength, and Achilles tendon (AT) stiffness. Dancers (training group *n* = 7, control group *n* = 7) performed MVIP at plantarflexed (10◦) and neutral ankle positions (0◦) followed by ramping isometric contractions equipped with ultrasound to assess strength and AT stiffness, respectively. Dancers also performed *saut de chat* leaps surrounded by 3-D motion capture atop force platforms to determine center of mass and joint kinematics and kinetics. The training group then followed a 12-week ankle-focused program including isometric, dynamic constant external resistance, accentuated eccentric loading, and plyometric training modalities, while the control group continued dancing normally. We found that the training group's *saut de chat* ankle PP (59.8%), braking ankle stiffness (69.6%), center of mass PP (11.4%), and leap height (12.1%) significantly increased following training. We further found that the training group's MVIP significantly increased at 10◦ (17.0%) and 0◦ (12.2%) along with AT stiffness (29.6%), while aesthetic leaping measures were unchanged (peak split angle, mean trunk angle, trunk angle range). Ankle-specific block progression training appears to benefit *saut de chat* leaping performance, PP output, ankle-joint kinetics, maximal strength, and AT stiffness, while not affecting kinematic aesthetic measures. We speculate that the combined training blocks elicited physiological changes and enhanced neuromuscular synchronization for increased *saut de chat* leaping performance in this cohort of dancers.

## Introduction

Progressive strength and conditioning programs that elicit increased power output are implemented in ballistic athletes to enhance stretch-shortening cycle (SSC) function (Cormie et al., 2010; Haff and Nimphius, 2012). As knowledge on the benefits of resistance exercise continually grows, additional training in ballistic athletes such as dancers, has become increasingly prevalent within dance companies, schools, and teams (Farmer and Brouner, 2021). Although the perception is shifting, optimal programming for dancers is still in the developmental stages (i.e., exercises, volume, intensity, and rest). For additional training to be effective, the following core questions must be addressed: (1) what is the overarching athletic goal, (2) what physical characteristics should be developed to achieve this goal, and (3) what are the known training modalities that will achieve these physical characteristics to achieve the overarching athletic goal? The pre-existing training volume dancers undergo from a young age must be considered when creating a strength and conditioning program. Furthermore, practitioners must be mindful of dancers' extreme range of motion and the need to preserve flexibility. While some researchers have accounted for training volume, range of motion, and flexibility, there is a lack of consideration for sport-specific training in dancers; that is, to model after dance-specific movements to further improve or expand physical attributes which enhance performance of dance-specific movement.

*Saut de chat* (split leap) performance is a fundamental skill in dance styles such as ballet, jazz, lyrical, and contemporary. Healthy dancers experience up to ~3.4 × bodyweight of force during the unilateral take-off portion of a *saut de chat* (Jarvis and Kulig, 2016). To perform a *saut de chat*, dancers typically *chassé*, or take two steps, prior to a unilateral take-off. At take-off, dancers will *developpé*, or flex, the leading leg up to hip level into a full split aerial position (ideal would be 180°; see **Figure 3**). Throughout the entire leap, dancers are instructed to maintain posture wherein the hips are directly beneath the shoulders to achieve aesthetic appeal. The nature of this postural maintenance is recognized as an aesthetic constraint (described in further detail in a topical perspective Rice and Nimphius, 2020). That is, to leap as high (or as far) as possible while achieving a full split position, dancers must rely less on hip flexion and subsequent torque generation. Likely due to the aesthetic constraint, ankle power is reportedly higher than knee and hip power during the take-off phase of a *saut de chat* (Jarvis and Kulig, 2020). The importance of the ankle during leaping is further supported by recent findings where dancers with greater plantar flexion strength and whole body center of mass peak power output leap higher as well as *better* aesthetically (Rice et al., 2021). It was also found that medial gastrocnemius and Achilles tendon stiffness predicted leap height (Rice et al., 2021). Although limited data exists on isolated joint training, implementing additional training for dancers that emphasizes developing strength, power, and muscle-tendon properties, specifically about the ankle joint, may elicit improved dance-specific SSC performance like the *saut de chat*.

Isolated joint training, while seemingly too specific, is gaining traction among strength and conditioning practitioners to hone in on either weakness or strength of a joint's surrounding tissues (Baltich et al., 2014; Rajic et al., 2020; Rice and Nimphius, 2020). In the context of dancers and leaping, isolated ankle-joint training may serve a three-fold purpose: (1) to enhance dance-specific SSC performance (Rice et al., 2021), (2) to prevent injury (Moita et al., 2017), and (3) to maintain or increase aesthetic appeal (Brown et al., 2007). While it appears that multi-joint exercise interventions may still benefit dancers' athletic and qualitative dance-specific performance (Angioi et al., 2012; Dowse et al., 2020; Escobar Alvarez et al., 2020; Grigoletto et al., 2020), isolated ankle-joint exercises might elicit adaptations that directly translate to *saut de chat* leaping biomechanics while preserving aesthetics (Rice and Nimphius, 2020). Previous research demonstrates that increasing maximal strength, rate of force development, muscle cross-sectional area, tendon stiffness, joint stiffness, and joint power concomitantly result in improved SSC performance (Kyrolainen et al., 2005; Kubo et al., 2007; Lamas et al., 2012; Katsikari et al., 2020; Laurent et al., 2020). As is well-established in the strength and conditioning field, not one training regimen results in all the aforementioned training adaptations. Because of this, dancers might benefit from an isolated ankle-joint block progression training program that incorporates a variety of training modalities as opposed to a singular training style.

Block progression training is the organization of different training modalities in a successive fashion that assists in performance realization through phase potentiation (Suchomel et al., 2018). For example, the adaptations that occur from isometric training and plyometric training have shown to differ from one another (Kubo et al., 2017); however, both styles of training progress athletic development in different ways. When ordering the different training modalities, practitioners should consider the temporal aspect of adaptations elicited from each respective training modality. Most importantly, the final block of training should help to collectively realize physical adaptations for the overarching athletic goal to be accomplished. For dancers, the following sequence might best influence *saut de chat* leaping performance: isometric, dynamic constant external resistance (DCER), accentuated eccentric loading (AEL), and plyometric training modalities. It has been proposed that isometrics can be divided into two sub-categories: pushing/pulling and holding (Schaefer and Bittmann, 2017). Holding and *balancing* a pre-determined load, as opposed to exerting maximal force against a stationary resistance, likely requires greater specificity of neuromuscular control strategies (Schaefer and Bittmann, 2017). Thus, holding isometrics may tap into motor unit recruitment synchronization (feedback driven), whereas pushing/pulling isometrics might increase high threshold motor unit recruitment and maximum activation (central command driven) (Pucci et al., 2006; Jeon et al., 2020). Traditional strength training, referred to here as DCER (involving both eccentric and concentric muscle actions), has been studied from several aspects of neuroplasticity and strength development for improved athletic capabilities (Aagaard et al., 2002). For dancers, loading full range of motion exercises is crucial, particularly about the ankle-joint, to potentiate force-generating capabilities across a spectrum of joint angles (transferring to a *plié* prior to leaping) allowing for greater power output during SSC actions (Taber et al., 2016). Some known physiological effects of AEL are increased tendon stiffness, muscle fiber CSA, and number of sarcomeres in series (Vogt and Hoppeler, 2014). AEL additionally serves as a means of Achilles tendinopathy “*pre-habilitation*” (O'Neill et al., 2015), which is highly prevalent in dancers. Plyometric exercises, involving the intention to move as quickly as possible, overload the eccentric phase of a SSC and necessitate optimal muscle-tendon interaction during the transition (amortization) phase into the concentric phase (Hirayama et al., 2017). Due to several confounding factors, plyometric training effects vary, but have most consistently shown to increase SSC performance in some fashion (i.e., jump height, power output, joint stiffness, and joint moments) (Kyrolainen et al., 2005; Cormie et al., 2009; Hirayama et al., 2017).

Dancers tend to specialize much earlier than other team sport athletes and therefore generally have less experience with other types of exercise. Due to this, the prescribed stimulus is especially important for dancers; whose diverse choreography necessitates mixed training. The purpose of this study was to investigate the effect of an ankle-focused block progression training program (24 sessions) on *saut de chat* leaping performance, maximal plantar flexion strength, and Achilles tendon stiffness. Specifically, we sought to measure center of mass and joint kinetics and kinematics to identify biomechanical and aesthetic aspects of leaping performance. We hypothesized that the training group would significantly increase *saut de chat* leaping performance (leap height, center of mass peak power, ankle peak power, and braking ankle stiffness), maximal plantar flexion strength, and Achilles tendon stiffness after training compared to the control group.

## Materials and Methods

### Experimental Design

To determine the effect of 12-weeks of ankle-specific block progression training, we assessed maximal strength, Achilles tendon stiffness, and *saut de chat* leaping performance in dancers. Dynamometry was used to assess maximal voluntary isometric plantar flexion strength at two ankle angles. Dynamometry as well as ultrasonic techniques were used to assess Achilles tendon stiffness. Reflective markers were placed on the dancers, and they completed six *saut de chat* leaps atop five force platforms surrounded by nine motion capture cameras. Relative center of mass peak power, leap height (center of mass displacement), ankle power, knee power, hip power, braking ankle stiffness, peak split angle, average trunk angle, and trunk angle variation were calculated from leaping trials. The training group then followed a 12-week ankle-specific block progression program including isometric, DCER, AEL, and plyometric training modalities while the control group continued dancing normally. Our contention was that dancers (whom already train 6+ h per week) may benefit from the previously described adaptations in each block progression for ankle-specific movements (Kanehisa et al., 2002; Haff and Nimphius, 2012; Suchomel et al., 2018). Block 1 (isometrics) was intended to enhance joint-specific strength at critical ranges of motion (Kanehisa et al., 2002; Kubo et al., 2006) and neuromuscular control. Block 2 (DCER) projected to continue strength enhancements with full range of motion dynamic exercises containing low-to-moderate repetition ranges. Block 3 (AEL) was anticipated to increase load tolerance related to power development (Haff and Nimphius, 2012). Lastly, block 4 (plyometrics) was programmed to maximize phase potentiation effects for SSC performance translation (Bohm et al., 2015). Thus, the residual training effects of the previous training blocks were intended to cooperatively prepare athletes for plyometric training that would subsequently result in improved *saut de chat* leaping performance. The overall structure of the blocks was intended to induce tendon remodeling, increase maximal strength, and improve ankle-specific SSC performance. Following the training, all testing measures were repeated to determine whether there was a training effect on muscle-tendon properties and dance-specific SSC performance.

### Experimental Procedures

A convenience sample size of dancers (*n* = 14) with a minimum of 10 years of ballet and jazz, modern, contemporary, or lyrical training that were dancing three or more times a week volunteered for the training group (*n* = 7; training age = 19.9 ± 5.6 year) or the control group (*n* = 7; training age = 19.4 ± 5.0 years). Due to some participants' schedules, the intervention was not feasible time-wise in addition to dance training, school, work, etc. The authors acknowledge that without random allocation, some level of bias may exist. The training group completed a 12-week ankle-specific block progression training program and dancing, while the control group continued dancing normally without additional training. Exclusion criteria required that participants reported no lower leg injuries within the previous 6 months, neuromuscular disease, or previous resistance training experience. Prior to data collection, the University Ethics Committee (#21229) approved all procedures. We sent an information letter to participants prior to arrival outlining all procedures.

Upon arrival, participants signed written informed consents and filled out a medical screening questionnaire. After we measured height and body mass, we performed whole body Dual-energy X-ray (DXA) scans (Hologic, Discovery A, Waltham, MA) to determine subject-specific lower limb segment masses for 3D motion capture kinematic and kinetic calculations. An operator positioned participants for the DXA scan while participants laid supine (Hart et al., 2015).

**Figure 1:**
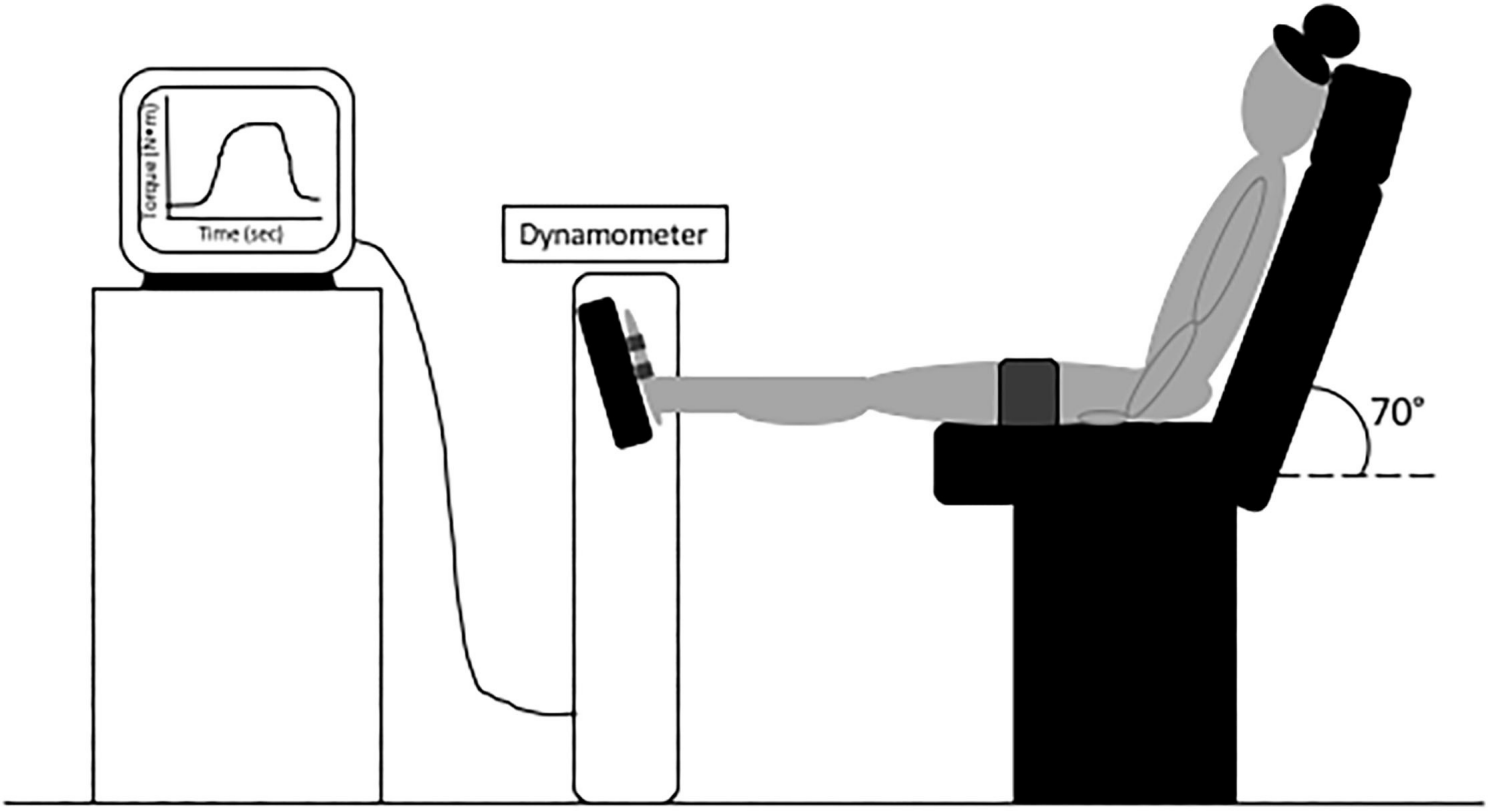
Experimental set-up to perform maximal voluntary isometric plantar flexion and isometric ramping contractions with dynamometry.

Participants then reported which leg was their preferred leaping leg (i.e., which leg would be leading) to determine the take-off leg that would be tested in maximal strength and tendon stiffness assessments with dynamometry (Biodex System 4, Biodex Medical Systems, Shirley, New York). We calibrated the Biodex prior to each testing session. Participants sat with the hips at an angle of 70° (slightly extended) in the Biodex (Figure 1). The preferred take-off foot was strapped securely in a pedal attachment with additional athletic tape. Furthermore, participants were moved to a position where the knee was slightly flexed at resting so that no heel lift occurred when the knee was fully extended. The testing leg was adjusted to be aligned directly in front of the hip both vertically and horizontally in the Biodex. To test maximal voluntary isometric plantar flexion (MVIP) at a slightly plantarflexed position, the ankle angle was moved to +10°. Participants then performed three MVIP trials, plantarflexing as fast and as hard as possible. We verbally encouraged participants and allowed 2 min of rest between each MVIP.

**Figure 2:**
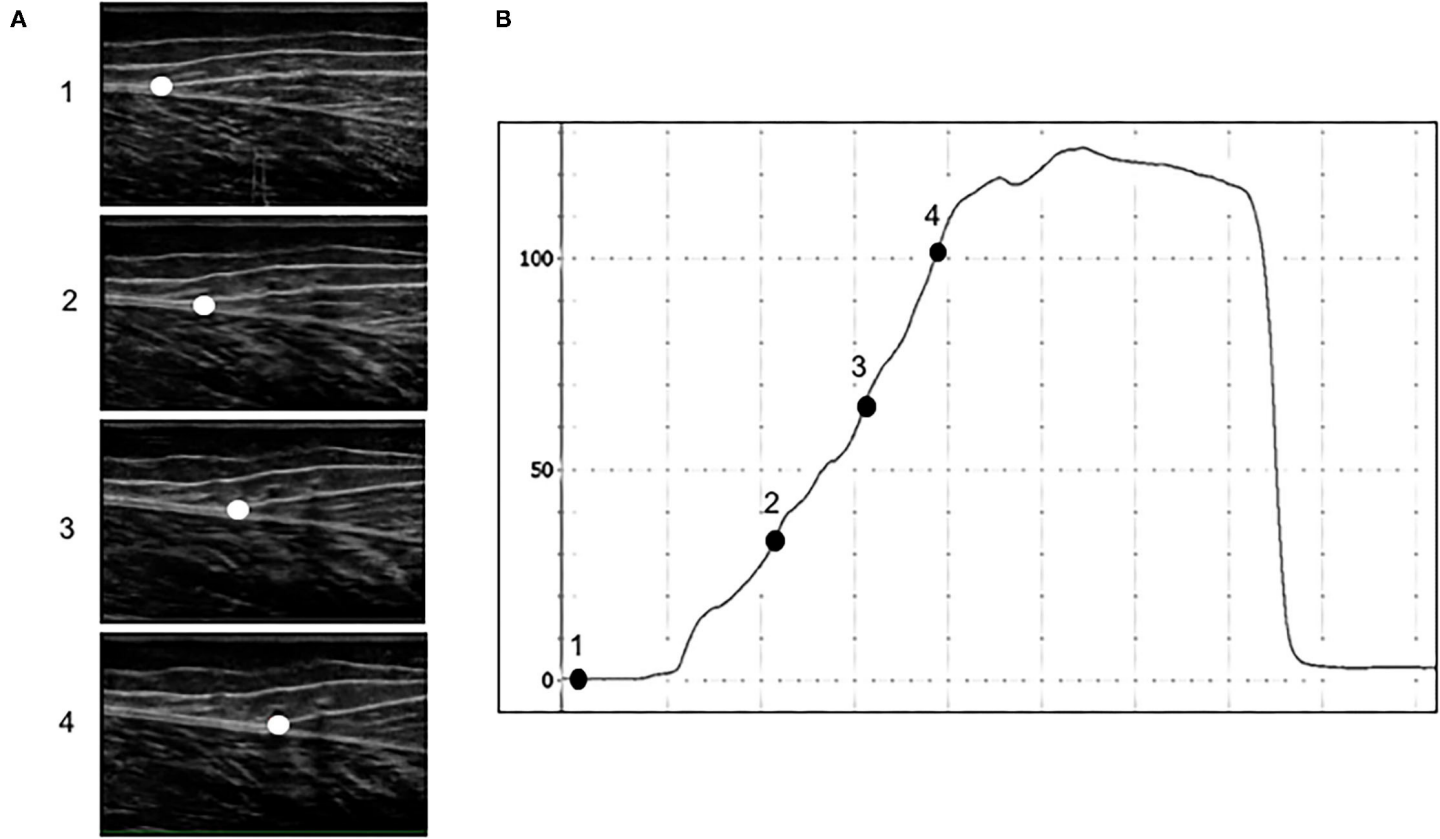
An example of a participant's ramping isometric contraction at the medial gastrocnemius and Achilles tendon junction **(A)** ultrasound images and **(B)** torque-time curve during (1) resting, (2) 25%, (3) 50%, and (4) 80% of peak torque.

The ankle was then moved to a neutral position (0°), and an ultrasound probe (ProSound F75, Hitachi Healthcare Americas, Twinsburg, Ohio) with real-time imaging (5.0 MHz wave frequency with a 50-mm scanning length, 22 Hz) was secured to the skin at the gastrocnemius and Achilles tendon musculotendinous junction. Ultrasound data was synchronized with ankle torque output using LabChart software (version 8.1.5, ADInstruments, NSW, Australia) and a 16-bit analog to digital converter (PowerLab 16/35, ADInstruments, NSW, Australia). We again instructed participants to perform three trials of MVIP, each separated by 2 min of rest. After determining the highest peak torque from MVIP trials at a neutral ankle angle, we set visual feedback guidelines on a screen in front of participants ±10 N•m from the peak torque value. Participants performed three trials of ramping isometric contractions (Figure 2), wherein we asked that they isometrically plantarflexed for 3 s up to 100% of their target torque and steadily hold for 3 s before relaxing (McCrum et al., 2018). Each trial was separated by 90 s of rest. To obtain the triceps surae moment arm, we used a previously published digital photographic method (Pohl and Farr, 2010; Rice et al., 2017). Tendon force was calculated by dividing the peak torque at 25, 50and 80% of each ramping isometric contraction torque trial by the estimated triceps surae moment arm. We measured the corresponding tendon displacement at 25, 50, and 80% of peak torque from the initial resting position of each trial (Figure 2) with Tracker software (Version 5.1.4, https://physlets.org/tracker). Tendon forces and displacements were averaged across subjects and plotted against one another. Tendon stiffness for each participant was defined as the slope from 25 to 80%. The highest peak torque from the slightly plantarflexed (10°) and neutral (0°) MVIP trials was recorded for each dancer.

**Figure 3:**
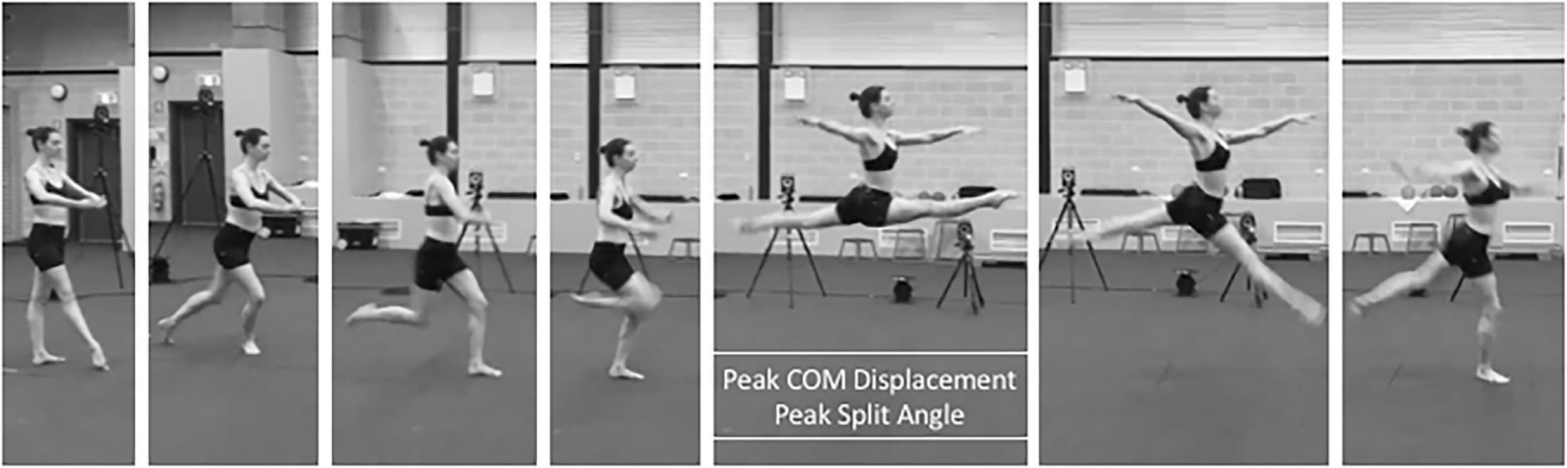
An example of a participant equipped with reflective markers surrounded by 3D motion capture performing a *saut de chat* leap over in-ground force platforms.

Participants then warmed up and stretched as they would normally to train, rehearse, or perform for the *saut de chat* leaps. We assessed *saut de chat* performance with three-dimensional motion capture to determine joint kinematics and kinetics with nine cameras (ViconMX F20, Vicon, Oxford, UK, 250 Hz sampling rate) surrounding five force platforms (900 × 600 mm, 9287CA and 9287BA, Kistler, Winterthur, Switzerland, 1,000 Hz sampling rate) beneath flooring (Mondo Sp.p.A., Alba, Italy). The motion capture system was calibrated prior to each data collection. We equipped dancers with 38 reflective markers (9.5 mm) in accordance with the UWA lower limb model (Chinnasee et al., 2018). Markers were adhered to the skin with double-sided tape on the trunk (C7, T10, clavicular notch, xiphoid process of sternum), hips (left and right anterior iliac spine, left and right posterior iliac spine), thighs (clusters of four), knees (right and left medial and lateral femoral condyles), lower legs (clusters of four), ankles (right and left medial and lateral malleoli), and right and left metatarsophalangeal and 5th metatarsal joints. For joint center determination and axes per the UWA kinematic and kinetic model, participants stood on a force platform surrounded by the cameras and performed a right leg “swinger” trial, a left leg “swinger” trial, five squats, and a static trial (Besier et al., 2003). Dancers then completed 2–3 familiarization leaps prior to data acquisition to a metronome of 106 beats per min (Jarvis and Kulig, 2016). We instructed dancers to take two steps, to leap as high as possible, and maintain arms in third position during the leap (one arm in the sagittal plane and one arm in the frontal plane, each at shoulder-height) (Figure 3; Wyon et al., 2013). Dancers performed a total of six *saut de chat* leaps.

We processed three-dimensional motion capture data with Nexus software (Version 2.11.0, Oxford, UK) and filtered marker trajectories with a 12 Hz cut-off frequency using a zero-lag low pass Butterworth 4th order filter. The three leaps with the highest whole body center of mass displacement (leap height), calculated from the UWA lower limb model output in Nexus, were selected for further analysis. The following leaping variables were analyzed with a custom-designed LabVIEW program (Version 19.0, National Instruments, Austin, TX): whole body center of mass peak power, joint powers, braking ankle stiffness, peak split angle, sagittal mean trunk angle and trunk angle range. Trials with insufficient marker data that were unable to calculate joint kinetics were removed from analysis. Marker data was deemed insufficient if gaps were unable to be filled as joint powers were unable to be computed. This resulted in a training group *n* = 7 and control group *n* = 5 for all pre- to post-testing joint kinetic measures. Forward dynamics were used to calculate velocity from force data. The initial center of mass position was corrected to maintain dynamic consistency since dancers were dynamic upon force platform contact (Rice et al., 2021). Relative center of mass peak power was calculated as the product of force and velocity. We obtained joint powers from the product of ankle-, knee-, and hip-joint moments and respective joint angular velocities during the take-off portion of the leap. Joint powers were normalized to body mass and relative concentric peak power was reported for each leaping trial. From here, relative joint power-time curves were re-sampled to 60 samples as was the average for all trials (the raw sample range was 49–75 samples). Pre-testing and post-testing average joint power-time curves were generated for training and control groups. Braking ankle stiffness was calculated from raw data as the slope of the ankle moment-angular velocity curve from force platform contact until the ankle angle began plantarflexing and contributing to propulsion. The braking ankle stiffness was then normalized to body mass. For aesthetic measures, peak split angle, mean trunk angle and trunk angle range were determined. Peak split angle was determined from summated front leg global femur and tibia angles and summated back leg global femur and tibia angles calculated in Nexus. We identified the time at which peak split angle was the highest for both front and back leg angles by adding all four segment angles together and dividing by two. We recorded sagittal mean global trunk angle and trunk angle range from Nexus from when force decreased <10 N during take-off and at the onset of >10 N of force during landing. For statistical purposes, the absolute value of mean trunk angles were compared among groups and testing sessions, however, the true means and standard deviations were reported.

### Training Intervention

**Figure 4:**
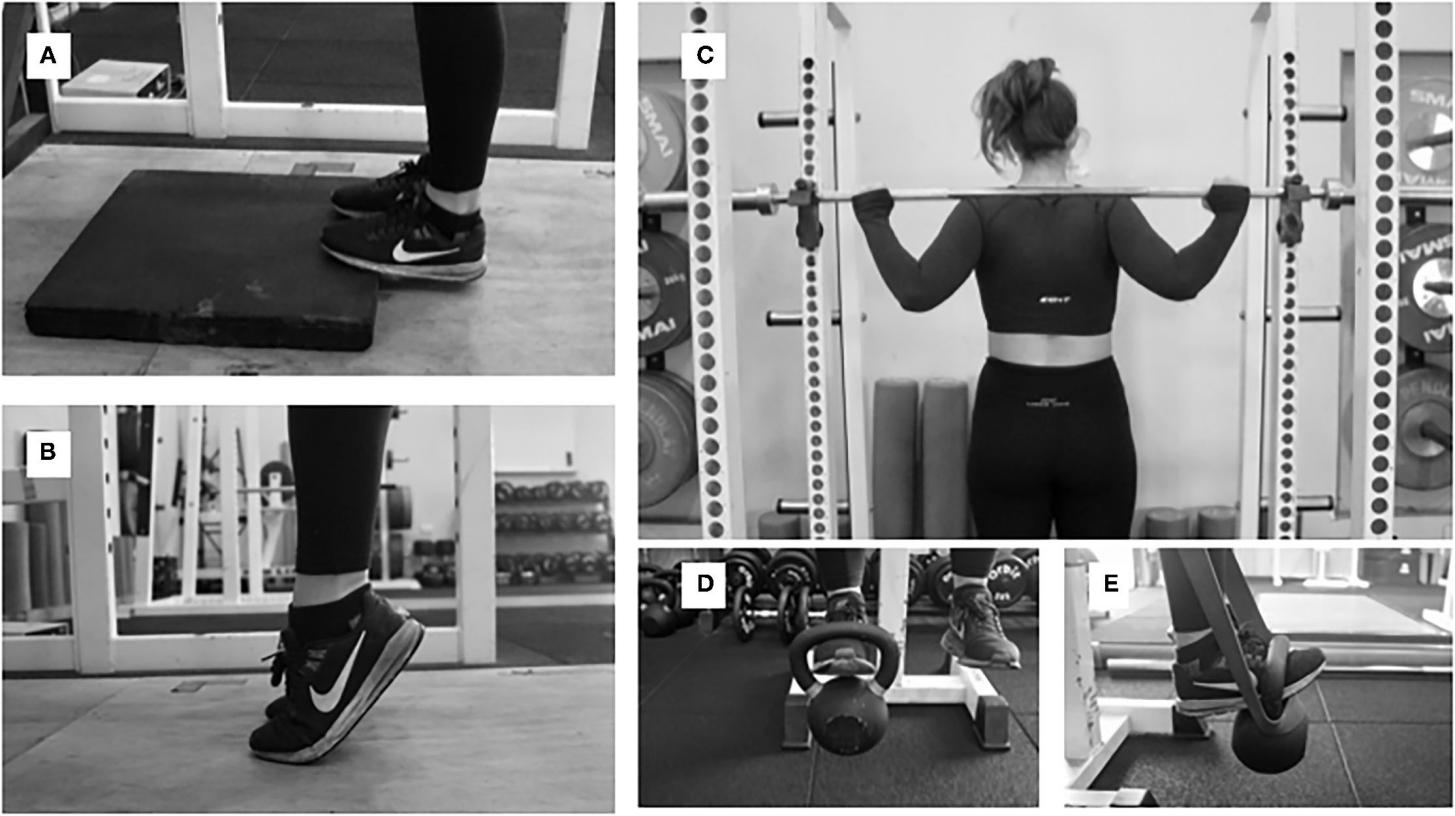
**(A)** Long muscle ankle position, **(B)** short muscle ankle position, **(C)** bar setup for “pushing” isometrics, **(D)** ankle position for “holding” dorsiflexor isometrics with a kettlebell, and **(E)** band and kettlebell setup for DCER and AEL of dorsiflexor muscles.

**Figure 5:**
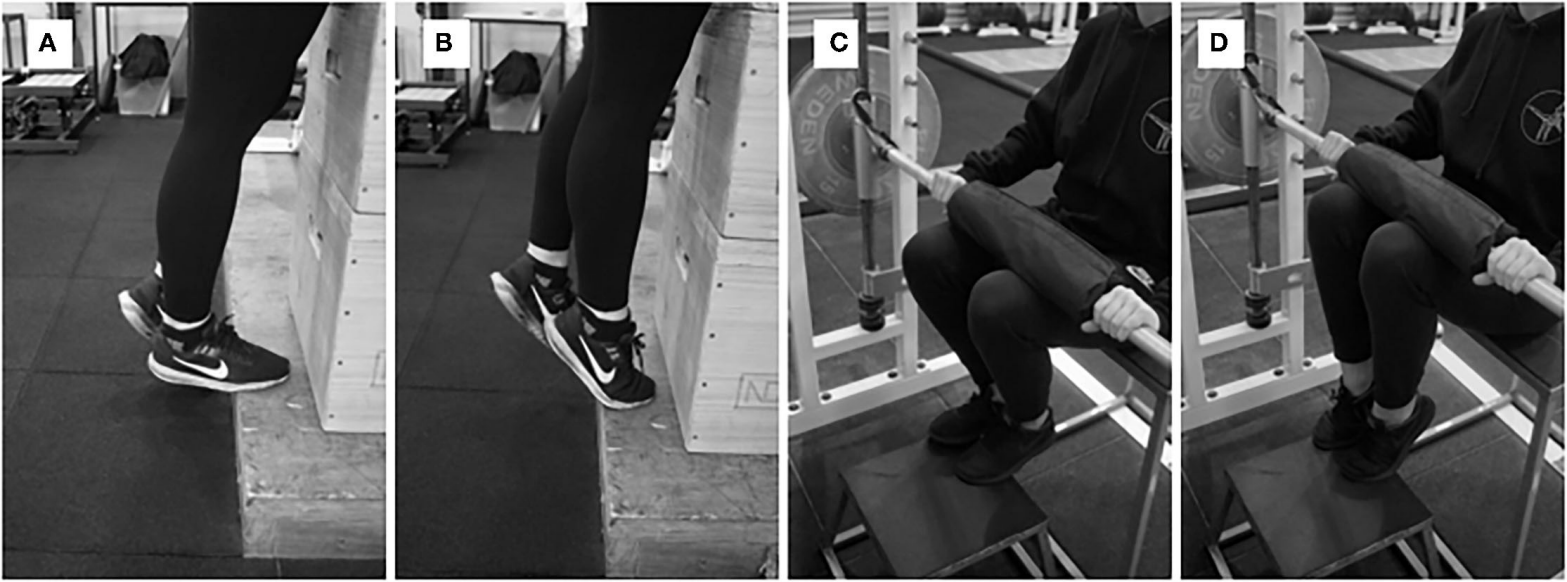
**(A,B)** Single-leg plantar flexion raises with performed with a dumbbell, and **(C,D)** single-leg seated plantar flexion with a Smith machine.

**Figure 6:**
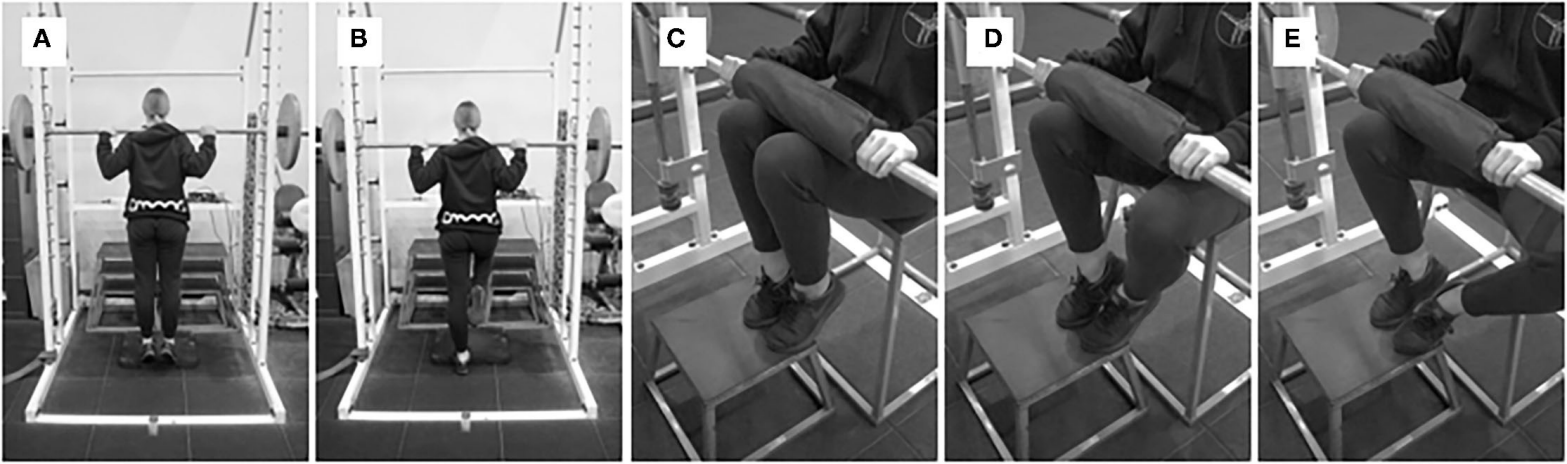
**(A,B)** Standing AEL off plantarflexor muscles, and **(C–E)** seated AEL of plantarflexor muscles.

**Figure 7:**
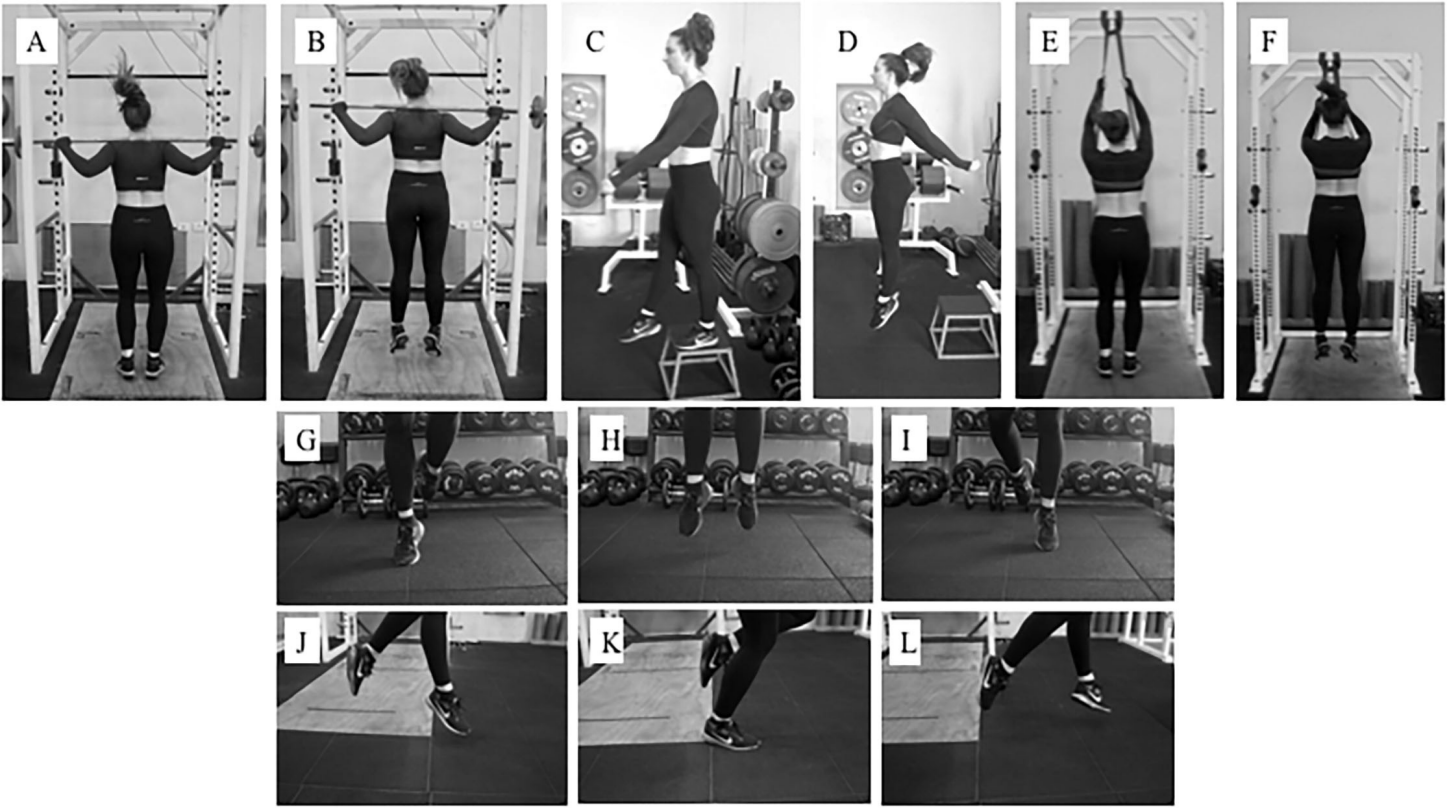
**(A,B)** Barbell hopping, **(C,D)** Box drop hops, **(E,F)** Unloaded band drops, **(G–I)** Side-to-side hops, and **(J–L)** Single-leg forward bounds.

After pre-testing, we instructed the control group to continue dancing normally. The training group attended two 30–45 min training sessions per week with the lead investigator (insert initials) for 12 weeks. Table 1 lays out the ankle-specific multi-targeted block progression training program we implemented including four blocks: isometrics, dynamic constant external resistance, accentuated eccentric loading, and plyometrics (Rice and Nimphius, 2020). Prior to each workout, participants would complete a warm-up consisting of 10 bodyweight squats, 10 calf raises, 10 band dorsiflexions, and 10 band ankle eversions/inversions. To determine proper loading, dancers completed a one-repetition MVIP with the ankles at a neutral position standing atop a portable force platform with an immovable bar across the shoulders. The highest peak force from three trials was used to determine certain loads during the 12-weeks of training (see Table 1; Figures 4–7). We implemented a constant volume for each block but a progressive intensity for the first 3 weeks of each block followed by a downloading week that overlapped with the successive block. Once training concluded, participants rested for 1 week prior to commencing post-testing, which was identical to pre-testing.

**Table 1:** Twelve weeks of isolated ankle-joint block progression training including sets, repetitions, inter-repetition rest, inter-set rest, intensity, and tempo of block exercises.

	**Sets × reps**	**Inter-rep rest**	**Inter-set rest**	**Intensity**	**Tempo**
**Isometrics (Weeks 1–4)**	3 × 3 s; “	3 s; “	60 s; “	85; 90; 95; 85%/ push/pull as hard as possible	0.3.0; “
Exercises	PF hold (L. muscle; Sh. muscle)	PF Push (L. muscle; Sh. muscle)	KB DF hold (neutral)	DF pull (L. muscle; Sh. muscle)	
**DCER (Weeks 4–7)**	4 × 6; “	—	90 s; “	85; 90; 95; 85%	1.1.1
Exercises	PF w/BB	Unilat. PF w/DB	Seated PF	DF w/KB and band	Ev/inversion w/band
**AEL (Weeks 7–10)**	3 × 6; “	—	90 s; “	130; 135; 140; 130%	3.1.1
Exercises (SM)	Standing PF	Seated PF	DF w/KB and band		
**Plyometrics (Weeks 9–12)**	4 × 6; “	—	1:10 work: rest; “	30; 35; 40; 30%	As fast as possible
Exercises	BB hops	Box drop hops	Unloaded band hops	SL side-side hops (med ball)	SL forward bounds (med ball)

*PF, plantar flexion; DF, dorsiflexion; KB, kettlebell; L., long; Sh., short; BB, barbell; Unilat., unilateral; DB, dumbbell; Ev, eversion; SM, smith machine; SL, single-leg; “: the following weeks were the same as the initial week*.

### Statistical Analysis

We performed a two-way factorial repeated measures analysis of variance (ANOVA) to identify within-subject effects and between-subject effects of training on group and time in the following variables: Achilles tendon stiffness, MVIP peak torque at 0 and 10°, leap height, relative peak power during leaping, relative braking ankle stiffness, relative ankle peak power, relative knee peak power, relative hip peak power, sagittal mean trunk angle, and sagittal trunk angle range. Prior to performing each two-way repeated measure ANOVA, data was inspected for outliers and a Levene's Test of Equality of Variances to determine whether equal variance existed in the data between groups and testing sessions. For the average joint power-time curves, we performed an exploratory analysis using a multivariate ANOVA to determine whether group or time effects existed. We expressed results as mean ± SD. We additionally calculated mean differences (μd), 95% confidence intervals (CI), and Hedge's *g* effect sizes. Hedge's *g* effect sizes were interpreted as trivial (<0.25), small (0.25–0.50), moderate (0.5–1.0), and large (>1.0) (Rhea, 2004). Statistical analyses were performed using SPSS software (version 25.0, SPSS Inc., Chicago, IL, USA). We used Pillai's trace to test overall significance, which was set *a priori* at *P* ≤ 0.05. We reported partial eta squared ($\eta_{\text{p}}^{2}$) for main and interaction effects, interpreted as small (0.01), medium (0.06), and large (0.14) (Cohen, 1992). Individual two-tailed Student's *T*-Tests were calculated if Pillai's trace indicated a significant effect of group, time, or group × time.

## Results

### Anthropometry

For anthropometric measures between groups, no significant differences existed, and no significant changes occurred between pre-testing and post-testing (Table 2).

**Table 2:** Anthropometric at pre- and post-testing sessions for the training and control groups.

**Group**	**Age (yrs)**		**Height (m)**		**Body mass (kg)**	
	**Pre**	**Post**	**Pre**	**Post**	**Pre**	**Post**
Training	24.86 ± 6.26	25.14 ± 5.98	1.62 ± 0.05	1.62 ± 0.05	58.38 ± 6.08	59.44 ± 7.33
Control	23.29 ± 4.39	23.71 ± 4.39	1.65 ± 0.04	1.65 ± 0.04	63.44± 10.10	63.40± 10.73

*Values are shown as means ± SD*.

### Maximal Voluntary Isometric Plantar Flexion Strength

**Figure 8:**
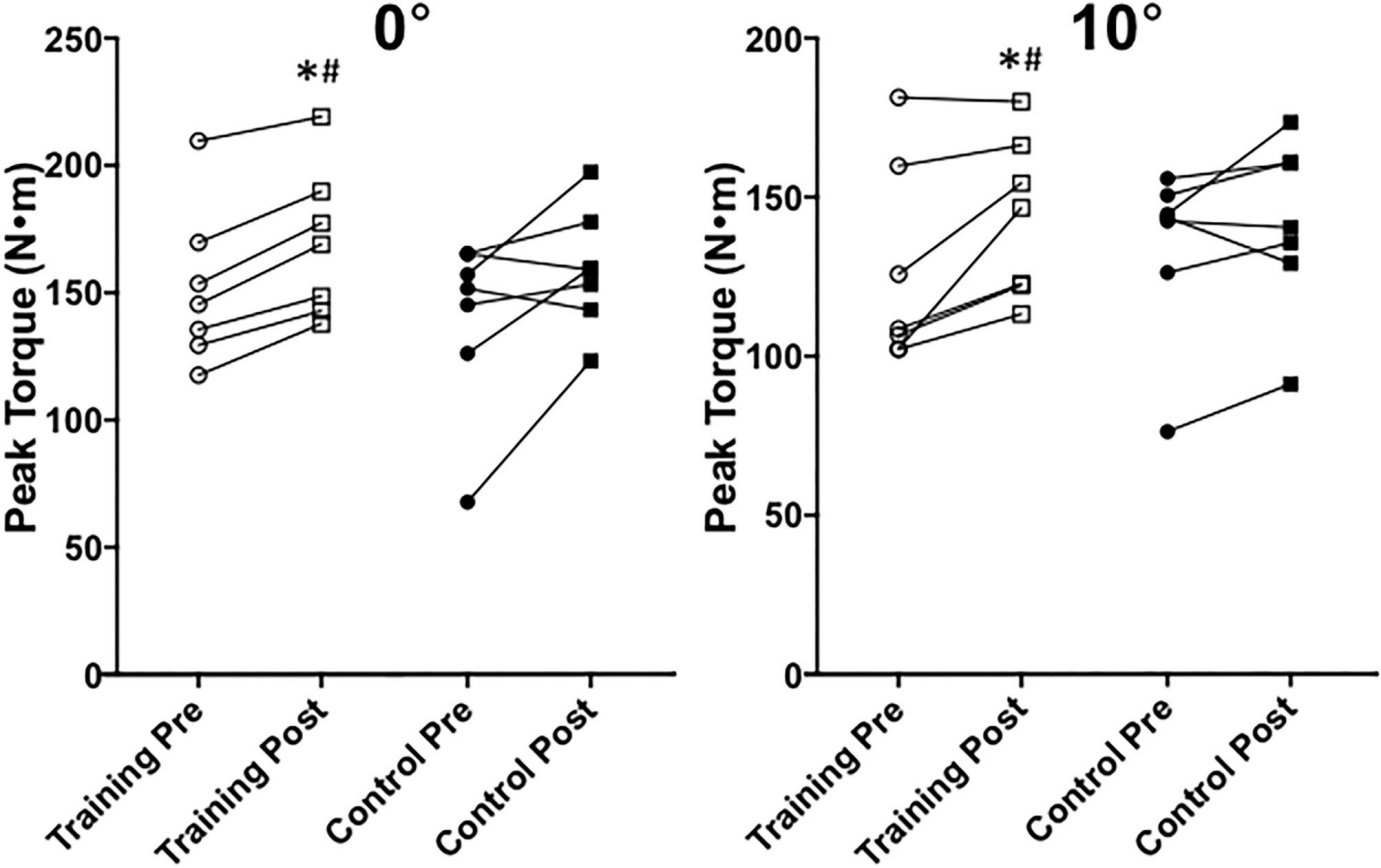
Maximal voluntary isometric plantar flexion peak torque at 0◦ (neutral) and 10◦ (slightly plantarflexed) between training and control groups at pre- and post-testing sessions. *Indicates a significant (*P* ≤ 0.05) training difference between pre- and post-testing sessions for the training group. ^#^Indicates a significant (*P* ≤ 0.05) difference between training and control groups at post-testing. Values are shown as individual data.

A significant group effect existed between subjects (*P* = 0.04, $\eta_{\text{p}}^{2}$ = 0.44) and a significant time effect existed within subjects (*P* = 0.005, $\eta_{\text{p}}^{2}$ = 0.62) for 10 and 0° MVIP peak torque. Ensuing Univariate tests demonstrated that there was a significant time effect for 10° MVIP peak torque (*P* = 0.007, $\eta_{\text{p}}^{2}$ = 0.47) as well as 0° MVIP peak torque (*P* = 0.001, $\eta_{\text{p}}^{2}$ = 0.61). We found that maximal strength between the training and control groups was not significantly different at pre-testing (10°: *P* = 0.22; 0°: *P* = 0.10). At post-testing, the training group possessed significantly higher MVIP peak torque at 10° (*P* = 0.05, μd = 37.65 ± 41.63 N; CI = 20.52 to 72.94; *g* = 1.54) and 0° (*P* = 0.04, μd = 42.08 ± 53.34 N; CI = 9.51 to 89.16; *g* = 1.60) than the control group (Figure 8). Figure 8 also demonstrates that the training group significantly increased MVIP peak torque at 10° (*P* = 0.03, μd = 17.01 ± 15.13 N; CI = 3.01 to 31.00; *g* = 0.56) and 0° (*P* < 0.0001, μd = 17.69 ± 5.61 N; CI = 12.50 to 22.88; *g* = 0.55) from pre- to post-testing. The control group's maximal strength did not significantly change from pre- to post-testing at either ankle angle (10°: *P* = 0.20; 0°: *P* = 0.08).

### *Saut de Chat* Leap Height and Peak Power

**Figure 9:**
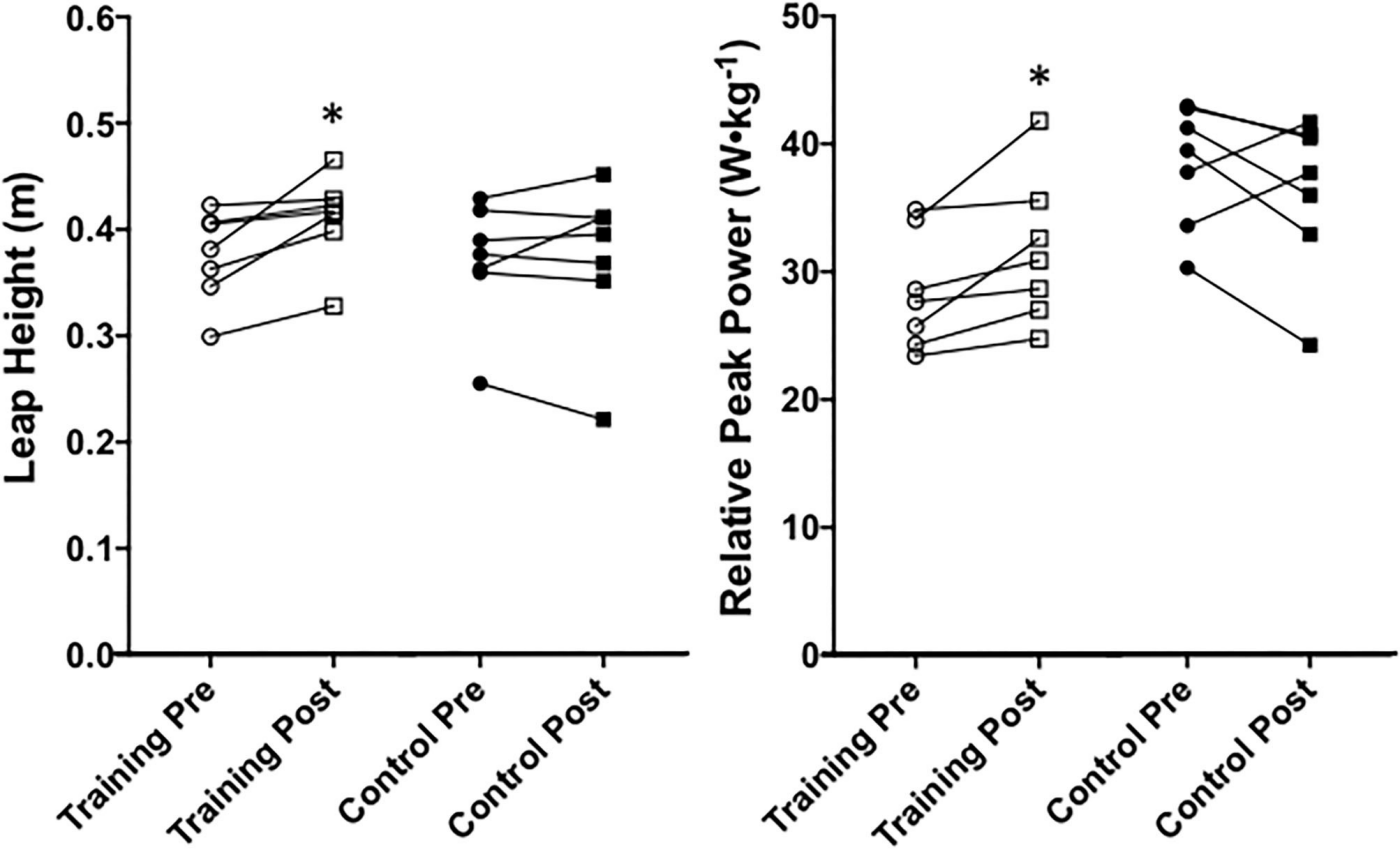
Leap height and relative peak power of the center of mass during *saut de chat* leaping between training and control groups at pre- and post-testing sessions. *Indicates a significant (*P* ≤ 0.05) training difference between pre- and post-testing sessions for the training group. Values are shown as individual data.

A significant time x group effect was found for leap height (*P* = 0.05, $\eta_{\text{p}}^{2}$ = 0.29). The training group leaped significantly higher at post-testing than at pre-testing (*P* = 0.02, μd = 0.036 ± 0.030 m; CI = 0.008 to 0.063; *g* = 0.79), and the control group remained unchanged (*P* = 0.95) pre- to post-testing. No significant differences existed between groups at pre-testing (*P* = 0.85) or post-testing (*P* = 0.26) for leap height. A significant group x time effect (*P* = 0.02, $\eta_{\text{p}}^{2}$ = 0.40) was found for relative peak power. The training group significantly increased relative peak power during leaping from pre-testing to post-testing (Figure 9; *P* = 0.01, μd = 3.23 ± 2.87 W•kg^−1^; CI = 0.58 to 5.89; *g* = 0.51). The control group's relative peak power did not significantly change from pre-testing to post-testing (*P* = 0.09) and was not significantly different from the training group at pre-testing (*P* = 0.97) or post-testing (*P* = 0.98).

### *Saut de Chat* Leap Joint Kinetics

**Figure 10:**
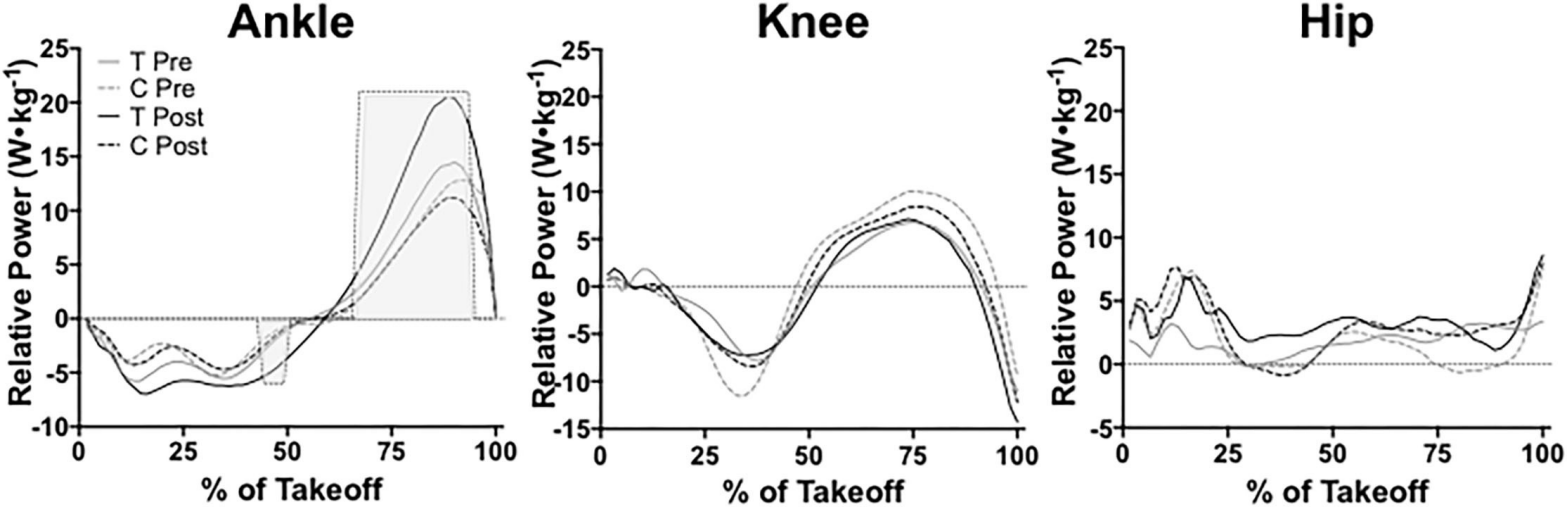
Ankle, knee, and hip power during the entire take-off portion of leaping between training (T) and control (C) groups at pre- and post-testing sessions. Light gray shading indicates a significant (*P* ≤ 0.05) difference between groups at post-testing. Values are shown as means.

We found no significant main effects between groups at pre-testing for ankle power (*P* = 0.13, $\eta_{\text{p}}^{2}$ = 0.997). At post-testing, we found a significant group effect for ankle power between training and control groups (*P* = 0.02, $\eta_{\text{p}}^{2}$ = 0.999). Ankle power differed significantly between groups from 44 to 49% and 67 to 93% of the leap take-off (shown with shading in Figure 10). Ankle power remained unchanged from pre- to post-testing in the training group (*P* = 0.50, $\eta_{\text{p}}^{2}$ = 0.96) and the control group (*P* = 0.28, $\eta_{\text{p}}^{2}$ = 0.98). At pre-testing, groups did not significantly differ from one another in knee power (*P* = 0.95, $\eta_{\text{p}}^{2}$ = 0.66). Knee power did not significantly differ between training and control groups at post-testing either (*P* = 0.51, $\eta_{\text{p}}^{2}$ = 0.95). Knee power also remained unchanged from pre- to post-testing for the training group (*P* = 0.77, $\eta_{\text{p}}^{2}$ = 0.88) and the control group (*P* = 0.99, $\eta_{\text{p}}^{2}$ = 0.44). Hip power did not significantly differ between groups at pre-testing (*P* = 0.36, $\eta_{\text{p}}^{2}$ = 0.98) or post-testing (*P* = 0.23, $\eta_{\text{p}}^{2}$ = 0.99). From pre- to post-testing, hip power remained unchanged for the training group (*P* = 0.76, $\eta_{\text{p}}^{2}$ = 0.89). The control group's hip power also remained unchanged pre- to post-testing as well (*P* = 0.67, $\eta_{\text{p}}^{2}$ = 0.88).

We found a significant time x group effect for ankle peak power (*P* = 0.04, $\eta_{\text{p}}^{2}$ = 0.35), but not for knee peak power (*P* = 0.26, $\eta_{\text{p}}^{2}$ = 0.13) or hip peak power (*P* = 0.53, $\eta_{\text{p}}^{2}$ = 0.04). Ankle peak power did not significantly differ between the training group and control group at pre-testing (*P* = 0.62), however, the training group possessed significantly higher ankle peak power at post-testing than the control group (*P* = 0.01, μd = 10.15 ± 3.16 W•kg^−1^; CI = 2.91 to 17.39; *g* = 1.77). The training group also significantly increased ankle peak power from pre-testing to post-testing (*P* = 0.04, μd = 6.51 ± 6.35 W•kg^−1^; CI = 0.64 to 12.39; *g* = 1.28) shown in Table 3. The control group's ankle peak power did significantly change from pre- to post-testing (*P* = 0.49). We found a significant time x group effect for relative braking ankle stiffness (*P* = 0.04, $\eta_{\text{p}}^{2}$ = 0.37). Braking ankle stiffness significantly increased in the training group from pre- to post-testing (*P* = 0.03, μd = 2.34 ± 2.24 W•kg^−1^; CI = 0.27 to 4.40; *g* = 1.25), but not the control group (*P* = 0.37). No differences existed for braking ankle stiffness between groups at pre-testing (*P* = 0.10) or post-testing (*P* = 0.23).

**Table 3:** Braking ankle stiffness, relative ankle, knee, and hip peak power between training and control groups at pre- and post-testing sessions.

**Group**	**Ankle stiffness (N·rad^−1^·kg^−1^)**		**Ankle peak power (W·kg^−1^)**		**Knee peak power (W·kg^−1^)**		**Hip peak power (W·kg^−1^)**	
	**Pre**	**Post**	**Pre**	**Post**	**Pre**	**Post**	**Pre**	**Post**
Training	3.80 ± 1.08	5.25 ± 1.44^#^	14.96 ± 4.64	20.56 ± 4.47^*^^#^	8.66 ± 3.24	8.31 ± 4.15	6.85 ± 2.89	6.50 ± 2.17
Control	5.87 ± 2.15	5.57 ± 2.32	13.98 ± 3.98	13.27 ± 5.93	10.42 ± 4.88	8.96 ± 2.67	6.09 ± 2.07	6.17 ± 1.57

* *Indicates a significant (P ≤ 0.05) training difference between pre- and post-testing sessions for the training group.*

# *Indicates a significant (P ≤ 0.05) difference between training and control groups at post-testing.*

*Values are shown as means ± SD*.

### *Saut de Chat* Leap Kinematics

We did not find a significant time x group effect for peak split angle during leaping (*P* = 0.23, $\eta_{\text{p}}^{2}$ = 0.12). Aesthetic trunk angle variables did not have a significant between time x group effect (*P* = 0.48, $\eta_{\text{p}}^{2}$ = 0.12) shown in Table 4.

**Table 4:** Aesthetic leaping variables (leap height, peak split angle, mean trunk angle, and trunk angle range) at pre- and post-testing sessions for training and control groups.

**Group**	**Leap height (m)**		**Peak split angle (°)**		**Mean trunk angle (°)**		**Trunk angle range (°)**	
	**Pre**	**Post**	**Pre**	**Post**	**Pre**	**Post**	**Pre**	**Post**
Training	37.48 ± 4.27	41.85 ± 4.10^*^	164.46 ± 6.35	164.55 ± 8.84	−5.17 ± 4.23	−4.76 ± 2.06	11.62 ± 1.72	12.34 ± 2.30
Control	37.79 ± 5.75	37.72 ± 7.59	163.45 ± 13.28	159.91 ± 15.25	−2.21 ± 4.02	−1.87 ± 5.26	7.71 ± 3.16	9.23 ± 3.72

* *Indicates a significant (P ≤ 0.05) training difference between pre- and post-testing sessions for the training group.*

*Values are shown as means ± SD*.

### Achilles Tendon Stiffness

**Figure 11:**
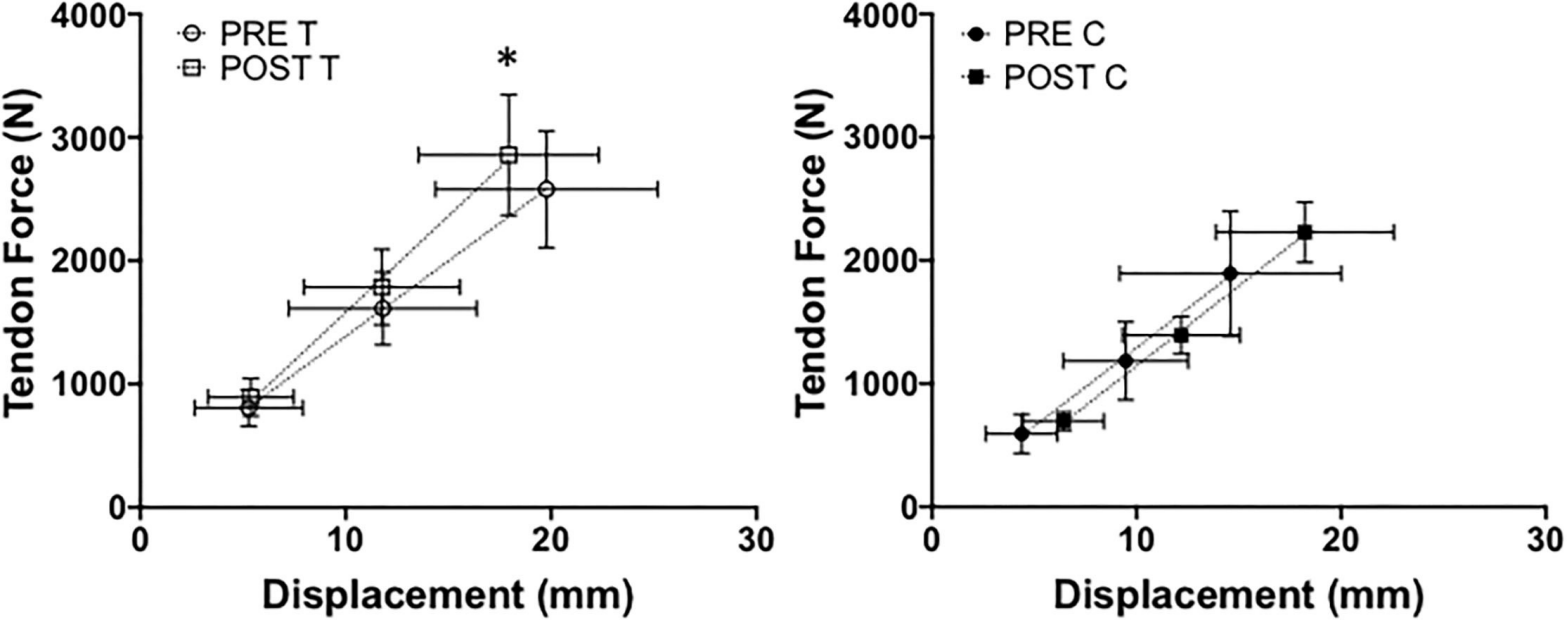
Achilles tendon stiffness between training (T) and control (C) groups at pre- and post-testing sessions. *Indicates a significant (*P* ≤ 0.05) training difference between pre- and post-testing sessions for the training group. Values are shown as means ± SD.

Significant group x time interaction was found for Achilles tendon stiffness (*P* < 0.0001, $\eta_{\text{p}}^{2}$ = 0.68) shown in Figure 11. Tendon stiffness did not differ between groups (*P* = 0.79, $\eta_{\text{p}}^{2}$ = 0.006) at pre-testing (*P* = 0.51; PreT = 128.73 ± 45.49 N•mm^−1^; PreC = 142.94 ± 41.60 N•mm^−1^) or post-testing (*P* = 0.14; Post-T = 163.85 ± 52.44 N•mm^−1^; Post-C = 136.90 ± 35.49 N•mm^−1^). The training group significantly increased tendon stiffness from pre-testing to post-testing (*P* < 0.0001, μd = 35.12 ± 12.66 N•mm^−1^; CI = 23.41 to 46.83; *g* = 0.67) while the control group remained unchanged (*P* = 0.40).

## Discussion

This study assessed whether an isolated ankle-joint training program would influence muscle-tendon properties that contribute to dance-specific SSC function. The main findings of our study indicate that 12 weeks of ankle-specific block progression training for dancers significantly improves (1) *saut de chat* performance, (2) maximal plantar flexion strength, and (3) Achilles tendon stiffness, while not negatively affecting aesthetics (trunk and peak split angle variables). In particular, ankle peak power increased by an average of 59.8%, braking ankle stiffness increased by an average of 69.6%, *saut de chat* leap height increased by an average of 12.1%, and center of mass peak power increased by an average of 11.4%, after training. We speculate that our training progression (as was intended) improved maximal strength and tendinous tissue properties that translated into ankle kinetics during leaping, which likely contributed to enhanced overall *saut de chat* performance (Rice et al., 2021).

As hypothesized, *saut de chat* leaping mechanics significantly improved after 12 weeks of isolated ankle-joint training. We believe that this was due to greater availability and realization of muscular power surrounding the ankle joint, which also appeared in higher center of mass peak power. Our joint power-time curves were comparable to previously measured leaping joint kinetics, further supporting the hypothesis that the ankle contributes most to performance (Perry et al., 2019; Jarvis and Kulig, 2020). In order to further explore the roles of the ankle, knee, and hip during leaping, we computed correlations (for all participants) among joint peak power data and leap height prior to training (*n* = 12). We found a significant, strong relationship between ankle peak power and leap height (*P* = 0.01, *r* = 0.69), and insignificant relationships between knee peak power and leap height (*P* = 0.26, *r* = 0.36) and hip peak power and leap height (*P* = 0.38, *r* = −0.28). Jarvis and Kulig have similarly observed the ankle joint both absorbs and generates the greatest amount of mechanical energy during the take-off portion of a *saut de chat* (Jarvis and Kulig, 2020). The concomitant increases in leap height, braking ankle joint stiffness, and Achilles tendon stiffness further support the prominent role of the ankle during leaping. The authors acknowledge the low sample size and recognize that our results warrant further investigation with more participants. Nevertheless, unmistakable adaptations to leap performance and dynamic joint control occurred. We encourage dance practitioners to incorporate additional training for the ankle as a means of increasing ankle power and dynamic joint control during dance-specific SSC actions.

We demonstrated that isometric, DCER, AEL, and plyometric training significantly increased *saut de chat* leap height without affecting aesthetics (peak split angle, mean trunk angle, and trunk angle range). After strength, plyometric, and power training, previous research indicates that dancers significantly improve subjective dance performance (Brown et al., 2007; Girard et al., 2015; Dowse et al., 2020). In contrast, we elected to quantitatively report leap height, split angle, and trunk control to gauge the effect of our training program on aesthetic competency. We suspect the higher leap height was due to the multitude of targeted neuromuscular and mechanical musculotendinous adaptations (Suchomel et al., 2018), which manifested into greater neural drive and force-generating capabilities (Aagaard et al., 2002). To our surprise, training group participants provided feedback that one of the noticeable training effects (namely during isometrics), was improved balance. In support of this, Trajković et al. discovered that plantar flexion and dorsiflexion strength were predictors of postural balance in a large cohort of elite athletes (Trajkovic et al., 2021), suggesting that an increase in strength would simultaneously improve balance. At least one exercise required spinal loading with a barbell in each block of our program. While anecdotal, it may be that improved and maintained aesthetics occurred due to ankle-joint loading and the accessory requirement of postural maintenance during several exercises.

Maximal strength at 10 and 0° of plantar flexion increased on average by 17.0 and 12.2%, respectively (14.6% when averaged together), after block progression training. In dance styles such as ballet, jazz, and lyrical, both aesthetic appeal (subjective evaluation) and performance (greater impulse) are influenced by hyper-plantar flexion (Koutedakis and Jamurtas, 2004; Rice et al., 2018). Dancers have displayed that peak torque production at a slightly plantarflexed position better predicts *saut de chat* leaping peak power than at a neutral position (Rice et al., 2021). Thus, we believe that the increase we observed in dance-specific SSC peak power was a downstream effect of targeted full range of motion ankle strength and power development in our training program. Moss et al. found that a strong relationship existed between one-repetition maximum and maximal power with a 2.5 kg load, postulating that heavy resistance training concomitantly benefits lighter load performance (Moss et al., 1997), relevant to dancers. Other exercise intervention studies with dancers as participants have similarly found strength and power to improve with different training modalities, such as resistance and plyometric training (Brown et al., 2007; Angioi et al., 2012; Dowse et al., 2020), however, ankle strength and *saut de chat* peak power were not measured. Moreover, it has been suggested that lower strength levels may be associated with higher injury rates in dancers (Moita et al., 2017), which again incites these athletes to partake in some combination of additional resistance training.

We lastly found that Achilles tendon stiffness increased from training by 29.6% on average. Our increases are comparable with previous findings wherein the most robust increases in tendon stiffness resulted from isometric or eccentric loading regimens (see review Bohm et al., 2015). During both voluntary and involuntary muscle contractions, tendinous tissues deform in response to mechanical loading of the muscle. By manipulating frequency, intensity, and rate of strain, mechanotransduction cell signaling pathways can stimulate functional adaptation to occur in tendinous tissues (Lavagnino et al., 2003). Increasing tendon stiffness may amplify muscle power output based on greater resistance to deformation during SSC actions (Hirayama et al., 2017). Interestingly, dancers with varying tendinopathies have shown to exhibit altered joint kinetics during leaping, possibly due to poor technique subsequently affecting leap performance and injury pre-disposition (Fietzer et al., 2012; Shih et al., 2021). From a clinical perspective, AEL addresses some of the overuse and strength disparities observed in individuals suffering from tendinopathies (O'Neill et al., 2015). The authors would like to highlight that while AEL stimulates collagen fiber cross-linkage formation (Maffulli et al., 2012), nutrition status and sufficient rest to support tendon remodeling are equally critical for tendon health.

In conclusion, a 12-week block progression program including isometric, DCER, AEL, and plyometric training modalities effectively improves dancers' SSC performance *via* positive alterations in muscle-tendon properties. Our findings specifically indicate that isolated ankle-joint training for aesthetic athletes increases maximal plantar flexion strength and Achilles tendon stiffness that likely translate into increased *saut de chat* ankle peak power, braking ankle stiffness, center of mass peak power, and leap height. Similar to other effective training interventions (Aagaard et al., 2002; Brown et al., 2007; Cormie et al., 2009; Hirayama et al., 2017), we contend that joint-specific strength and power increases are advantageous to dancers from both a performance enhancement and an injury prevention lens (Rice and Nimphius, 2020). In agreement with preceding researchers' beliefs, dancers are a unique group of highly-specialized athletes that require additional training to supplement the volume and loads of training they experience (Koutedakis and Jamurtas, 2004). Ultimately, our ankle-specific block progression training program positively impacted sport-specific performance, which should be the primary focus of all strength and conditioning approaches.

### Limitations

While our findings present interesting data on the effects of isolated joint training for dancers, there are some limitations. The small sample size (*n* = 7 in both groups) necessitates reproduction of the results with a larger sample size to generalize the outcomes for both dancers and other athletes. Specifically, two “visual outliers” existed for leap height and relative leap peak power: a training participant that improved quite a bit and a control participant that worsened some. Although equal variance existed in our data, these individuals could have affected statistics. Future researchers might also seek to compare isolated ankle-joint training with a generic resistance training program to verify that isolated joint training has added benefits. Manipulation of the block progression training (i.e., training modalities, block order, exercises, volume, intensity, rest, and program duration) might help to optimize training for dancers to improve *saut de chat* leap performance. Lastly, it would be interesting to observe empirically whether postural balance changes with isolated ankle-joint training in dancers by measuring center of pressure variables. Although more research is always needed, our training regimen did appear to induce neuromuscular and musculotendinous adaptations that benefited the overall athlete.

### Practical Applications

Strength and conditioning practitioners working with highly specialized athletes should determine the types of appropriate movements and loads which will not only maintain already developed skills, but improve them as well. Aesthetic athletes require additional training, distinct from team sport athletes, to target joint-specific strength and power. We aimed to improve maximal plantar flexion strength, Achilles tendon stiffness, and *saut de chat* performance with a block progression program including isometric, DCER, AEL, and plyometric training modalities. By isolating the ankle-joint during additional training, dancers appear to successfully translate improved muscle-tendon properties and SSC function into dance-specific leaping performance. Given the near 20 years of dance training our participants possessed, enhancing their execution of existing movement strategy for improved performance is a difficult task to accomplish. We found that by employing additional training that targets ankle-specific stretch-shortening cycle, neuromechanical, and muscle-tendon unit development, dancers are capable of expanding on already well-engrained movement execution strategies. Strength and conditioning approaches specifically addressing sport-specific joint loads may benefit overall athletic prowess and should be further investigated. We hope that sport science and dance science practitioners alike will consider implementing strength and conditioning approaches that address sport-specific constraints, goals, and ultimately, individual athletes' needs to improve overall performance.

## Data Availability Statement

The original contributions presented in the study are included in the article/supplementary material, further inquiries can be directed to the corresponding author.

## Ethics Statement

The studies involving human participants were reviewed and approved by Edith Cowan University. The participants provided their written informed consent to participate in this study.

## Author Contributions

PR contributed to the study design, data acquisition, data analysis, initial writing of the manuscript, and editing of the manuscript. KN and SN contributed to the study design and editing/final preparation of the manuscript. All authors contributed to the article and approved the submitted version.

## Funding

This study was funded by a 2018 US National Strength and Conditioning Association Doctoral Grant (G1003733). PR was funded by an Edith Cowan University Higher Degree by Research Scholarship. SN is a member of the Australian Center for Research into Injury in Sport and its Prevention (ACRISP) at Edith Cowan University. ACRISP is one of the International Research Centers for the Prevention of Injury and Protection of Athlete Health supported by the International Olympic Committee (IOC). We declare the results of the study are presented clearly, honestly, and without fabrication, falsification, or inappropriate data manipulation.

## Conflict of Interest

The authors declare that the research was conducted in the absence of any commercial or financial relationships that could be construed as a potential conflict of interest.

## Publisher's Note

All claims expressed in this article are solely those of the authors and do not necessarily represent those of their affiliated organizations, or those of the publisher, the editors and the reviewers. Any product that may be evaluated in this article, or claim that may be made by its manufacturer, is not guaranteed or endorsed by the publisher.
